# The causal effects of genetically predicted alcohol consumption on endometrial cancer risk from a Mendelian randomization study

**DOI:** 10.1038/s41598-024-53926-z

**Published:** 2024-02-12

**Authors:** Jie Yang, Xiang Qu, An-jie Zheng, Fan Jiang, Hui Chang, Jin-ru zhang, Li-juan Yan, Peng Ning

**Affiliations:** Department of Oncology, Baoji Gaoxin Hospital, No.19, Gaoxin 4 Road, Gaoxin District, Baoji, 721000 Shaanxi Province China

**Keywords:** Alcohol consumption, Mendelian randomization, Endometrial cancer, Causal inference, Protective factors, Cancer epidemiology, Cancer

## Abstract

Endometrial cancer (EC) is a common gynecological tumor in females with an increasing incidence over the past few decades. Alcohol consumption has been linked to the occurrence of various cancers; However, epidemiological studies have shown inconsistent associations between alcohol consumption and EC risk. In order to avoid the influence of potential confounding factors and reverse causality in traditional epidemiological studies, we used a method based on genetic principles-Mendelian randomization (MR) analysis to test whether there is a causal relationship between alcohol consumption and EC. MR analysis was conducted using publicly available summary-level data from genome-wide association studies (GWAS). Fifty-seven single nucleotide polymorphisms (SNPs) were extracted as instrumental variables for alcohol exposure from the GWAS and Sequencing Consortium of Alcohol and Nicotine GWAS summary data involving 941,287 participants of European ancestry. SNPs for EC were obtained from the Endometrial Cancer Association Consortium, the Endometrial Cancer Epidemiology Consortium, and the UK Biobank, involving 121,885 European participants. The inverse variance weighted (IVW) method was used as the primary method to estimate the causal effect, and the MR-Egger regression and weighted median method were used as supplementary methods. Sensitivity analyses were conducted using the Mendelian Randomization Pleiotropy RESidual Sum and Outlier global test, MR-Egger intercept test, and leave-one-out analysis to evaluate the impact of pleiotropy on causal estimates. An increase of 1 standard deviation of genetically predicted log-transformed alcoholic drinks per day was associated with a 43% reduction in EC risk [odds ratio (OR) = 0.57, 95% confidence interval (CI) 0.41–0.79, *P* < 0.001]. Subgroup analysis of EC revealed that alcohol consumption was a protective factor for endometrioid endometrial cancer (EEC) (OR = 0.56, 95% CI 0.38–0.83, *P* = 0.004) but not for non-endometrioid endometrial cancer (NEC) (OR = 1.36, 95% CI 0.40–4.66, *P* = 0.626). The MR-Egger regression and weighted median method yielded consistent causal effects with the IVW method. The consistent results of sensitivity analyses indicated the reliability of our causal estimates. Additionally, alcohol consumption was associated with decreased human chorionic gonadotropin (HCG) and insulin-like growth factor 1 (IGF1) levels. This MR study suggests that genetically predicted alcohol consumption is a protective factor for EC, particularly for EEC, and this protective effect may be mediated through the reduction of HCG and IGF1.

## Introduction

Endometrial cancer (EC) is one of the most common gynecological cancers in women. According to global cancer statistics in 2020, approximately 420,000 new cases of EC are diagnosed each year^1^. Although the mortality rate of EC is decreasing, the incidence rate of EC has been increasing at a rate of 0.58% per year over the past 30 years^2^. Among the potential factors contributing to the increased risk of cancer, alcohol consumption has received significant attention. Alcohol consumption is one of the main causes of increased all-cause mortality and is associated with various health conditions^3^. Globally, approximately 700,000 new cancer cases are attributed to alcohol consumption each year, with women accounting for one-fourth of these cases, indicating a significant burden of female cancer due to alcohol consumption^4^. The relationship between alcohol consumption and EC has been controversial. A meta-analysis of seven cohort studies showed a J-shaped relationship between alcohol consumption and the risk of EC^5^. The study found that daily consumption of less than one drink was associated with a decreased risk of EC, while consumption of more than two drinks was associated with an increased risk of EC, but all confidence intervals (CI) included null values. Three other meta-analyses^6–8^showed no relationship between alcohol consumption and the risk of EC. However, all current studies are observational studies, and therefore, cannot avoid problems such as reverse causation and potential confounding factors, resulting in a low level of evidence. Although randomized controlled trials (RCTs) have a higher level of evidence and can verify causal relationships, these trials are difficult to conduct due to high requirements, strict controls, and ethical considerations. Therefore, there is an urgent need to design well-conducted studies to prove the causal relationship between alcohol consumption and the risk of EC.

Mendelian randomization (MR) uses genetic variations strongly associated with an exposure factor as instrumental variables (IVs) to infer causal relationships between exposure and outcome factors^9^. According to Mendelian genetics, the random allocation of parental alleles to offspring is equivalent to the randomization process in RCTs, making it less susceptible to traditional confounding factors. In addition, the inheritance of genetic variations from parents satisfies the temporality criterion, which can avoid reverse causality. Therefore, MR studies are considered similar to RCTs but are more cost-effective, and are mainly used to verify causal relationships between exposure factors and outcomes^10^. Currently, there are many MR studies on alcohol consumption and cancer, such as the finding that alcohol consumption increases the risk of lung cancer in MR analysis^11^, while drinking with meals can reduce the risk of lung cancer^12^. Alcohol consumption is a risk factor for colorectal cancer in Asian populations^13,14^, but not associated with the risk of colorectal cancer in European populations^11^. Other MR study results also indicate that alcohol consumption is not associated with the risk of breast cancer, ovarian cancer, or bladder cancer^11,15,16^.

In this study, we used MR to investigate whether there is a causal relationship between genetically predicted alcohol consumption and EC.

## Materials and methods

This study is reported strictly according to the Strengthening the Reporting of Observational Studies in Epidemiology Using Mendelian Randomization (STROBE-MR) standard^17,18^.

### Genetic instrumental variables related to alcohol consumption

**Figure 1:**
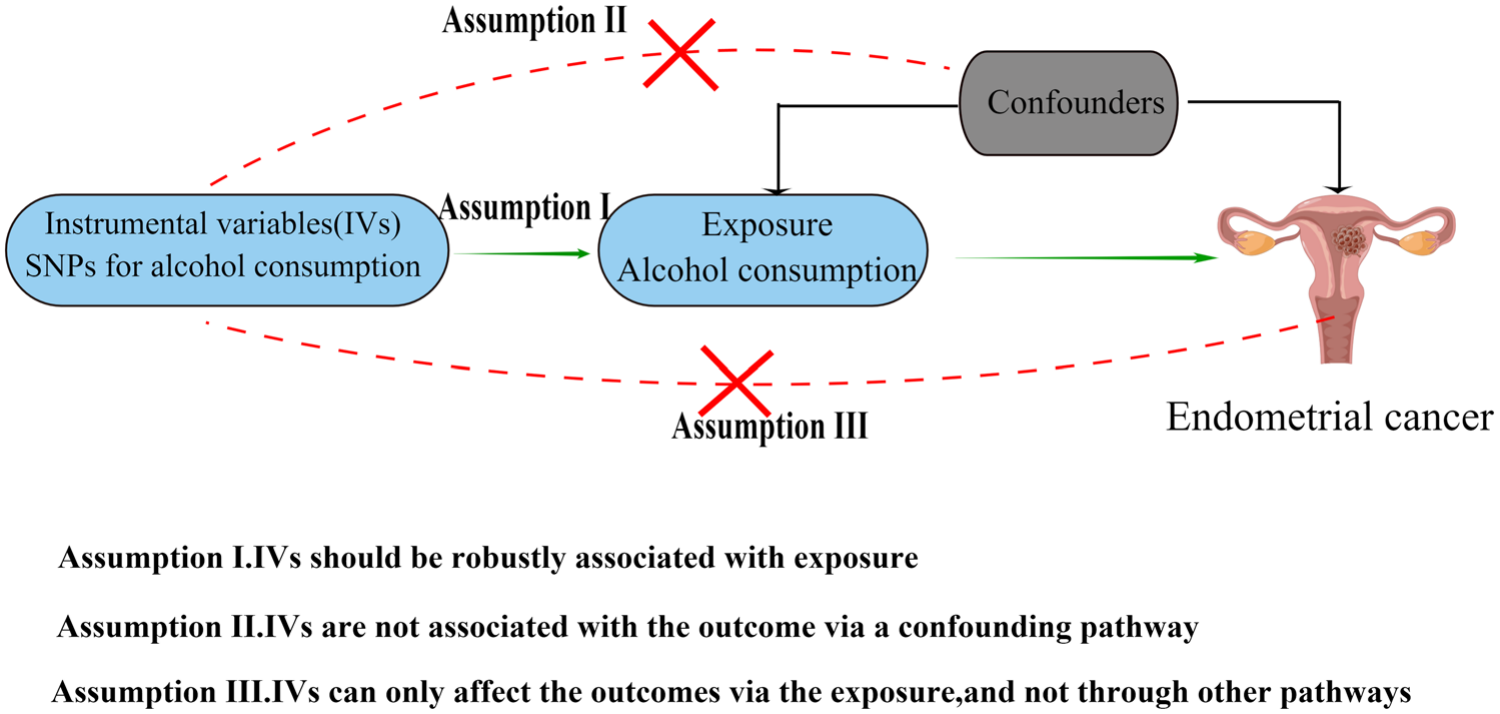
Key assumptions of the Mendelian randomization study.

We obtained SNPs related to log-transformed alcoholic consumption from the genome-wide association studies (GWAS) summary data of the GWAS and Sequencing Consortium of Alcohol and Nicotine (GSCAN), which involved 941,287 participants of European ancestry, including 403,939 individuals from the 23andMe dataset and 537,349 from 9 other datasets (excluding UK Biobank)^19^. The phenotype is based on responses to a single question in the Health Profile survey, "In the last two weeks, how many servings of alcohol did you drink each day? (1 serving equals 12 oz. of beer, 5 oz. of wine, or 1.5 oz. of hard alcohol)" with categorical responses "None" (= 0), "Between 0 and 1" (= 0.5), "1" (= 1), "2" (= 2), "3" (= 3), "4" (= 4), "5 or more" (= 7). The values were then transformed by $f\left(x\right)=\mathit{log}\left(x+e\right)$. As per the MR study design, the IV used to assess the causal relationship between alcohol consumption and EC should satisfy the following assumptions: (1) the IV should be strongly associated with alcohol consumption, (2) the IV should not be correlated with other potential confounding factors, and (3) the IV should not be directly associated with EC or only influence EC through alcohol consumption (Fig. 1). Here, SNPs from the GSCAN GWAS summary data were considered as potential IVs.

We obtained 88 independent SNPs as IVs to predict the daily log-transformed alcohol consumption by setting a significant threshold for the strong association between SNPs and alcohol consumption (*P *< 5 × 10^–8^) and removing linkage disequilibrium (r^2^ < 0.001 and distance > 10,000 kb). To satisfy hypothesis III, all selected SNPs were required to have no significant association with EC (*P *> 5 × 10^–5^). Additionally, to confirm whether these SNPs were causally related to EC through other phenotypes (i.e., confounding factors), we checked all SNPs by consulting previously published MR studies and using Phenoscanner V2 (http://www.phenoscanner.medschl.cam.ac.uk/). Confounding factors that were already known, including body mass index (BMI)^20,21^, basal metabolic rate (BMR)^22^, education level^23^, and low-density lipoprotein cholesterol (LDL-C) and high-density lipoprotein cholesterol (HDL-C)^24^, were excluded. Finally, we included 57 SNPs that were strongly associated with EC for further analysis, ensuring the satisfaction of MR hypotheses 2 and 3. To assess weak instrumentality, the F-statistic was calculated using the formula $F={R}^{2}(N-K-1)/K(1-{R}^{2})$, where R^2^ represents the proportion of variance in alcohol consumption explained by each SNP, K represents the number of SNPs extracted, and N represents the sample size of the GAWS study that is related to alcohol consumption^25^. A genetic variant with an F-statistic < 10 is usually considered a weak instrument^26^. The R^2^ value was calculated using the following formula: ${R}^{2}=2\times {(Beta)}^{2}\times EAF\times (1-EAF)/[2\times {\left(Beta\right)}^{2}\times EAF\times \left(1-EAF\right)+2\times {\left(SE\right)}^{2}\times N\times EAF\times (1-EAF])$, where EAF is the frequency of the effect allele, Beta is the effect size of the genetic variation on alcohol consumption, and SE is the standard deviation (SD) of the effect size of the genetic variation^27^ (Supplementary Table S1).

### EC in GWAS

Our EC GWAS data obtained EC-associated SNPs, which were provided by the Endometrial Cancer Association Consortium (ECAC), the Epidemiology of Endometrial Cancer Consortium (E2C2), and UK Biobank, comprising 12,906 cases and 108,979 population-matched controls of European ancestry^28^. EC was further classified by histological type, including 8,758 cases of endometrioid endometrial cancer (EEC) and 1,230 cases of non-endometrioid endometrial cancer (NEC).

There was no overlap between the exposed and outcome samples in this study. For the 57 SNPs associated with alcohol consumption, we obtained summary data from the aforementioned EC GWAS data (Supplementary Table S1). 

### Statistical analysis

We harmonized the alcohol dataset and the EC dataset by aligning the SNPs' allele directions and removing palindromic and incompatible SNPs. Fixed-effect inverse variance weighted (IVW-FE) meta-analysis was performed for individual SNPs' Wald ratios. The Wald ratio is the causal effect estimate for each SNP, calculated as the Beta in the outcome data divided by the same SNP's Beta in the exposure data. The inverse variance weighted (IVW) method is the primary approach for estimating the overall causal effect of exposure on the outcome, and different IVW models are selected based on the presence or absence of heterogeneity. The IVW method provides the most efficient causal relationship when all SNPs are valid IVs. However, even if one SNP is invalid, the results can still be biased. Therefore, the causal effect is estimated mainly by IVW method, supplemented by MR-Egger regression and weighted median method. The MR-Egger method corrects for pleiotropy and provides a causal effect estimate that is not biased by violating the IV assumption but has lower precision. The weighted median method provides a consistent causal effect estimate even if up to half of the weight comes from invalid SNPs.

In this study, in order to ensure the reliability of MR results, we used a variety of sensitivity analysis methods. Firstly, we use the Mendelian Randomization Pleiotropy RESidual Sum and Outlier (MR-PRESSO) method to detect and eliminate the horizontal multiple effects (outliers) of SNP in order to reduce the bias of causality^29^. Secondly, we use Cochran Q test to evaluate the consistency of each SNP estimate. If there is no significant heterogeneity, IVW-FE method is used; if there is significant heterogeneity, random effect IVW method (IVW-MRE) is used. Thirdly, we use MR-Egger intercept test to detect the existence of horizontal multiplicity. If horizontal multiplicity is found, the IVW method may not be suitable, so we use the weighted median method as the main analysis method^30^. MR-Egger intercept test and MR-PRESSO method can also help us to test whether the hypothesis of MR II and III is true. Finally, "Leave-one-out" analysis was used to assess the robustness of MR estimates by eliminating a different SNP in each iteration to quantify the causal influence of outlying SNPs and to ensure that deleting SNPs did not affect the MR estimates. Statistical power calculations were performed using an online tool (available at https://shiny.cnsgenomics.com/mRnd/ )^31^.

In addition, to investigate the potential mechanisms underlying the association between alcohol consumption and EC, we employed a MR approach to determine whether alcohol consumption affects established risk factors for EC. The mechanism by which alcohol consumption affects the risk of EC is unclear, it may be related to changes in hormone levels. Experimental study found that estrogen levels spike sharply for a short period of time after women drink alcohol^32,33^. A meta-analysis^34^ involving 200,000 women also showed that alcohol consumption is associated with increased concentrations of sex hormones [including estradiol (E2), estrone, androsterone, etc.], and EC is one of the most common hormone-dependent cancers. Therefore, we mainly studied several common hormone-related risk factors for EC, including E2^35^, human chorionic gonadotropin (HCG)^36^, insulin-like growth factor 1 (IGF1), sex hormone-binding globulin (SHBG)^35^, Total testosterone (TT) and membrane-associated progesterone receptor component 2 (PGRMC2)^36^. These GWAS summary data are available at the MRC IEU Open GWAS repository (https://gwas.mrcieu.ac.uk/) (Supplementary Table S2). In order to explore whether alcohol consumption mediates the risk of EC by affecting hormone levels, we continue to use 57 SNP from MR analysis of alcohol consumption and EC as tool variables for alcohol consumption, and perform MR analysis with hormone levels as the outcome. A series of sensitivity analyses were carried out to ensure the robustness of the results.

All MR analyses were performed using the “TwoSampleMR” and “MRPRESSO”packages in version 4.2.2 of R software.

## Results

### Causal relationship between alcohol consumption and EC

We ultimately included 57 alcohol-related SNPs in the analysis. The F-statistic for each individual instrumental variable and the overall F-statistic were both greater than 10, indicating no bias due to weak instruments in the study (Supplementary Table S1). With an odds ratio (OR) of 0.57 and a precision of 88% for EC, we obtained an unbiased causal estimate.

**Figure 2:**
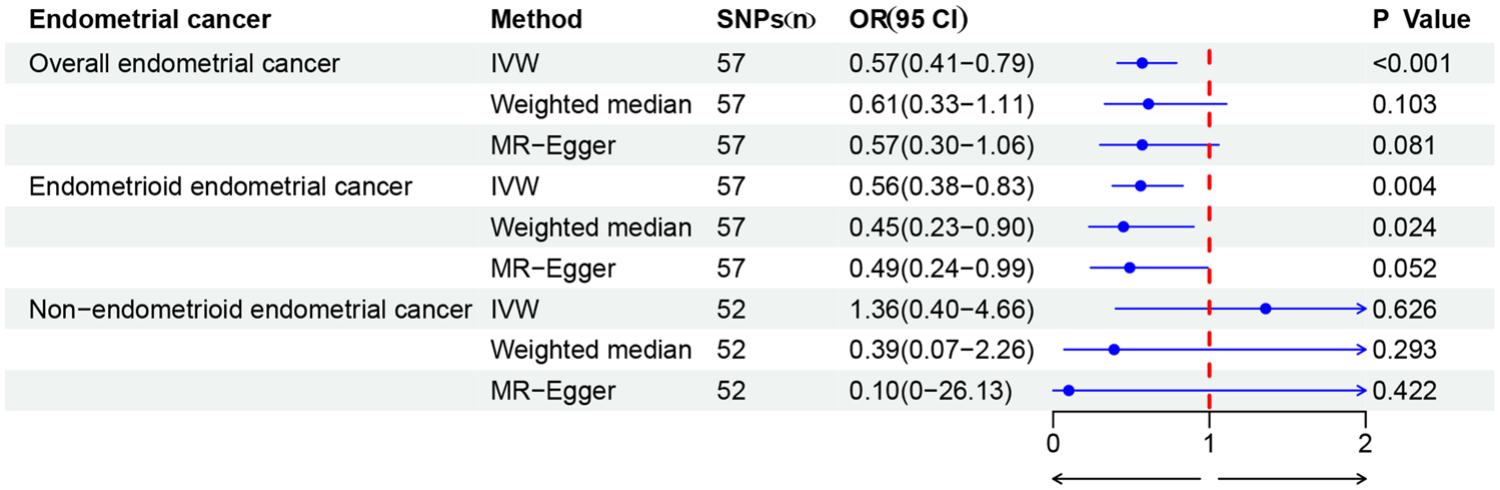
MR estimates from different methods of assessing the causal effect of alcohol consumption on EC.

**Figure 3:**
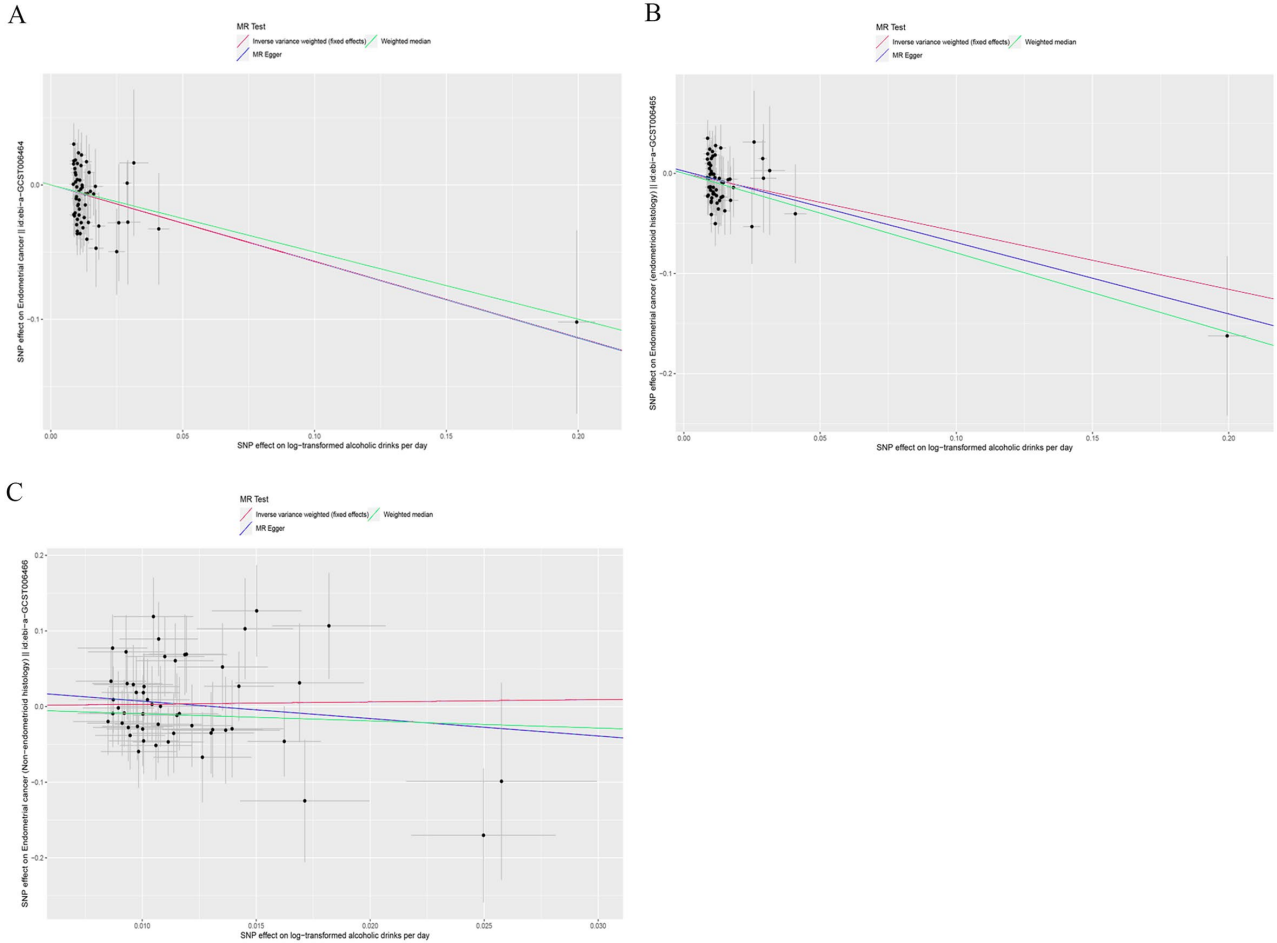
Scatter plots for Mendelian randomization (MR) analyses of the causal relationship between alcohol consumption and EC. (**A**) alcohol consumption-EC. (**B**) alcohol consumption-EEC. (**C**) alcohol consumption-NEC.

MR analysis showed that an increase of 1 SD in the natural log-transformed alcohol consumption per day was associated with a 43% reduction in EC risk (OR = 0.57, 95% CI 0.41–0.79, *P* < 0.001). The Weighted median method (OR = 0.61, 95% CI 0.33–1.11, *P* = 0.103) and the MR-Egger regression method (OR = 0.57, 95% CI 0.30–1.06, *P* = 0.081) also demonstrated consistent protective effects of alcohol consumption on EC (Fig. 2). The causal estimates for each SNP from the three MR methods are shown in Fig. 3 scatter plots.

**Figure 4:**
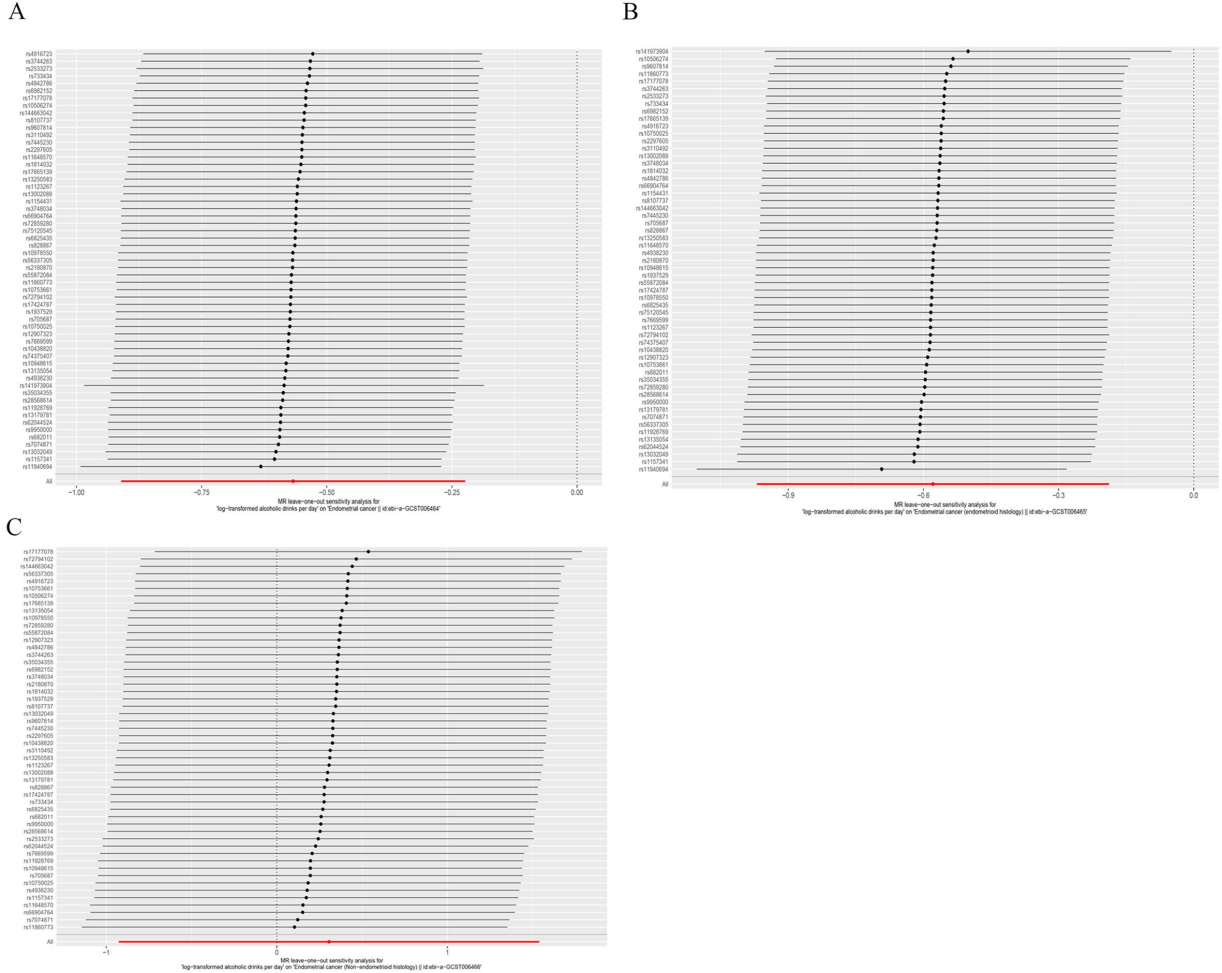
MR leave-one-out sensitivity analyses of the causal relationship between alcohol consumption and EC. (**A**) alcohol consumption-EC. (**B**) alcohol consumption-EEC. (**C**) alcohol consumption-NEC.

In sensitivity analysis (Table 1), the MR-PRESSO global test (*P* = 0.366) indicated the absence of outlier SNPs, and the MR-Egger intercept had a value of 3.75E−05 (*P* = 0.993), which suggested no evidence of significant pleiotropy in our causal estimates, meeting the assumptions 2 and 3 of MR. Our IVW method, Cochrane's Q statistic, was 59.97 (*P* = 0.334), indicating a low heterogeneity and reliable causal effect in our study. Finally, the leave-one-out sensitivity analysis also indicated that no outlier SNP affected the overall causal effect estimation (Fig. 4).

**Table 1:** Sensitivity analysis of alcohol consumption causally linked to endometrial cancer.

Endometrial cancer	MR-PRESSO test	Heterogeneity test		Horizontal pleiotropy		
	Global test *P* value	Q-statistics	P value	Egger-intercept	se	*P* value
Overall endometrial cancer	0.366	59.97	0.334	3.75E-05	0.0045	0.993
Endometrioid endometrial cancer	0.615	51.25	0.655	0.0023	0.0051	0.654
Non-endometrioid endometrial cancer	0.482	50.86	0.479	0.0302	0.0320	0.351

In the analysis of EC subtypes, the causal effect was only found in EEC (Fig. 2). It was observed that alcohol consumption was still a protective factor in EEC (OR = 0.56, 95% CI 0.38–0.83, *P* = 0.004), but was not associated with NEC (OR = 1.36, 95% CI 0.40–4.66, *P* = 0.626). Moreover, the Weighted median method and MR-Egger regression method also produced consistent results. The causal estimates for each SNP in the three MR methods for each subtype are shown in Fig. 3. In addition, a series of sensitivity analyses, such as the MR-Egger regression intercept test, MR-PRESSO, Cochrane's Q test (Table 1), and leave-one-out analysis (Fig. 4), demonstrated the robustness of the causal relationships in each subtype.

### Causal relationship between alcohol consumption and EC risk factors

MR analysis of the causal relationship between alcohol consumption and other risk factors for EC shows that alcohol consumption is a risk factor positively associated with E2 increase and a protective factor negatively associated with HCG and IGF1 decrease (Table 2). Although horizontal pleiotropy was present in the MR analysis of alcohol consumption and E2, the weighted median method provided consistent causal estimates even in the presence of horizontal pleiotropy. However, alcohol consumption has no causal relationship with SHBG, TT and PGRMC2. Sensitivity analysis of alcohol consumption and hormone MR is shown in Supplementary Table S3.

**Table 2:** Causal effect from alcohol consumption to endometrial cancer risk factors.

Outcomes	GWAS ID	Number of SNPs	Method	Causal effect	95% CI	*P* value
Estradiol	ebi-a-GCST90012105	55	Weighted median	1.03	1.00–1.06	0.034
Human chorionic gonadotropin	prot-a-688	57	IVW(MRE)	0.55	0.32–0.95	0.031
Insulin-like growth factor 1	ukb-d-30770_irnt	45	IVW(MRE)	0.88	0.80–0.98	0.017
Sex hormone-binding globulin levels	ebi-a-GCST90012111	57	IVW(FE)	1.02	0.99–1.04	0.207
Total Testosterone	ieu-b-4864	47	IVW(MRE)	1.08	0.99–1.17	0.680
Membrane-associated progesterone receptor component 2	prot-a-2260	57	IVW(MRE)	0.77	0.45–1.30	0.327

## Discussion

The results of this MR analysis indicate a strong causal relationship between genetically predicted alcohol consumption and EC risk. Specifically, an increase of one SD in log-transformed alcohol consumption per day was associated with a 43% lower risk of EC. Subgroup analyses showed that genetically predicted alcohol consumption had a protective effect only in the EEC subtype of EC, while the protective effect disappeared for NEC. In addition, we found that the protective effect of alcohol consumption on EC may be related to a decrease in HCG and IGF1.

Alcohol consumption is a biologically plausible cancer-promoting factor. Firstly, the ethanol metabolite acetaldehyde is recognized as a carcinogen, which can cause cancer in humans by binding to cellular proteins and DNA^37^. Secondly, alcohol consumption significantly increases postmenopausal women's estrogen levels^38^, which may increase the risk of estrogen-dependent cancers. Estrogen has long been considered a factor in the development of EC. However, there has been controversy about the relationship between alcohol consumption and EC risk, with numerous studies attempting to prove their relationship. Only a small amount of evidence suggests that alcohol consumption may increase the risk of EC. An Italian retrospective study found a positive correlation between alcohol consumption and EC risk^39^. Another prospective cohort study involving 41,574 participants of multiple races found that daily alcohol consumption of ≥ 2 drinks increased postmenopausal EC risk (RR = 2.01, 95CI%: 1.3–3.11) compared to not alcohol consumption^40^. Conversely, more evidence suggests a weak negative or no correlation between alcohol consumption and EC risk. A retrospective study in Japan found a negative correlation between alcohol consumption and EC risk in non-flushing women after drinking (P trend = 0.001)^41^. Conversely, this protective effect of alcohol consumption disappeared in patients who experienced flushing after drinking, which may be related to insufficient acetaldehyde dehydrogenase leading to an increase in acetaldehyde levels. A retrospective study in the United States also indicated a negative correlation between alcohol consumption and EC risk in obese women^42^. The NIH-AARP Diet and Health Study found a significant negative correlation between alcohol consumption and EC risk in women with a BMI ≥ 25 kg/m^2^ in a prospective cohort study involving 114,414 participants (*P* = 0.04)^43^. Another retrospective study found a significant negative correlation between moderate alcohol consumption and young (< 50 years old) women's EC risk^44^. A prospective study involving 68,067 participants from nurse health research showed that moderate alcohol consumption was associated with a 22% reduced risk of EC compared with non-drinkers^45^. Although prospective studies have less selection and recall bias than retrospective studies, they are still subject to bias due to potential confounding factors. For example, all studies did not adjust for dietary fiber intake, which has been reported to modify the association between alcohol consumption and estrogen-dependent cancers^46^. Additionally, we found that the protective effect of alcohol on EC was related to pathological type, and previous studies did not stratify by pathological type, which may also be a source of bias in the results. Researchers should be cautious when interpreting the results, as genetic variation has long-term effects on exposure levels, and the estimated values of MR studies are often larger than those of RCTs. Therefore, the causal estimates obtained from MR studies should not be directly interpreted as the direct effects of intervention in practice. Furthermore, although the study indicates that alcohol consumption can reduce the risk of EC, it can also lead to a series of other health problems.

The effect of alcohol on tumors may involve a variety of intermediate phenotypes. Although breast cancer and ovarian cancer are estrogen-dependent cancers, MR studies have not confirmed that alcohol consumption is associated with their risk^11,15^. This may suggest that alcohol consumption may activate some protective factors that counteract the effects of estrogen on these hormone-dependent tumors. E2 is a known risk factor for EC, which has been confirmed by the MR study^47^. Our study also found that alcohol consumption does increase E2 levels and increase the risk of EC, which is consistent with previous studies^34,38,48^. However, we also found that alcohol consumption can reduce HCG and IGF1 levels, this may be a relevant factor in reducing the risk of EC. HCG is related to endometrial proliferation and malignant tumor^49,50^. A cohort study of 677,247 women showed that increased HCG levels were significantly associated with EC (HR = 1.98, 95% CI 1.33–2.95)^51^. IGF1 receptors are widely distributed in endometrium^52^. IGF1 expression and signal transduction play an important role in the proliferation, secretion and menstrual cycle changes of premenopausal endometrium. IGF1 is also involved in the occurrence and development of EC^53,54^ and is related to the prognosis of EC^52^. In addition, IGF1 is also a downstream molecule of E2 and participates in the proliferation of EC cells^55^. There is evidence that alcohol consumption can lead to a decrease in IGF1 levels^48,56^, and our MR study also confirmed this.

### Advantages and limitations

Our study has several important advantages. Firstly, we conducted the first MR study to investigate the causal relationship between alcohol consumption and EC, which is a major advantage as it simulates an RCT and avoids reverse causation and potential confounding factors in observational studies. Secondly, we utilized Phenoscanner V2 and reviewed the literature to exclude SNPs with pleiotropic effects, meeting the three assumptions for MR analysis as much as possible. Additionally, given the large sample sizes in these studies and the robust alcohol-related instrumental variables (F statistics > 10), our study has sufficient statistical power (88%) to detect robust and precise causal effect estimates.

However, there are also some limitations. Firstly, our study used SNPs as alternative indicators for alcohol consumption, rather than directly studying the amount and frequency of alcohol consumption, which may have some measurement errors. Besides, SNPs as a phenotype for alcohol consumption may be influenced by gene environment interactions, which may lead to biased results. Secondly, because the study aggregated different types of alcoholic beverages, the effects of certain components, such as the flavonoids in red wine, were ignored. Therefore, it was not possible to distinguish whether different types of alcoholic beverages and drinking patterns have differential causal effects. Finally, since the study population consisted of European individuals, it may not be generalizable to other populations.

## Conclusion

The results of this MR study support a negative causal relationship between genetically predicted alcohol consumption and EC, which was only found in EEC, providing higher-level evidence for the long-standing association between alcohol consumption and EC risk. However, more evidence is still needed to support this conclusion.

### Supplementary Information

Supplementary Table S1.

Supplementary Table S2.

Supplementary Table S3.

Supplementary Table S4.

## Data Availability

Data used in the present study are all publicly available. GWAS summary data for alcohol consumption (Supplementary Table S4) were available in (https://www.ncbi.nlm.nih.gov/pmc/articles/PMC6358542/) and the remaining GWAS summary data (Supplementary Table S2 provides all GWAS ID) were obtained from the MRC IEU OpenGWAS repository (https://gwas.mrcieu.ac.uk/).

## Abbreviations

EC: Endomerial cancer
MR: Mendelian randomization
GWAS: Genome-wide association studies
SNPs: Single nucleotide polymorphisms
GSCAN: GWAS and Sequencing Consortium of Alcohol and Nicotine
IVW: Inverse variance weighted
MR-PRESSO: Mendelian Randomization Pleiotropy RESidual Sum and Outlier
SD: Standard deviation
OR: Odds ratio
CI: Confidence interval
EEC: Endometrioid endometrial cancer
NEC: Non-endometrioid endometrial cancer
E2: Estradiol
HCG: Human chorionic gonadotropin
IGF1: Insulin-like growth factor 1
SHBG: Sex hormone-binding globulin
TT: Total testosterone
PGRMC2: Progesterone receptor component 2
RCTs: Randomized controlled trials
IVs: Instrumental variables
BMI: Body mass index
BMR: Basal metabolic rate
LDL-C: Low-density lipoprotein cholesterol
HDL-C: High-density lipoprotein cholesterol
ECAC: Endometrial Cancer Association Consortium
E2C2: Epidemiology of Endometrial Cancer Consortium
IVW-FE: Fixed-effect inverse variance weighted
IVW-MRE: Random effects IVW method
